# Acknowledgement to Reviewers of *Biomolecules* in 2018

**DOI:** 10.3390/biom9010036

**Published:** 2019-01-21

**Authors:** Biomolecules Editorial Office

**Affiliations:** MDPI, St. Alban-Anlage 66, 4052 Basel, Switzerland

Rigorous peer-review is the cornerstone of high-quality academic publishing. The editorial team greatly appreciates the reviewers who have contributed their knowledge and expertise to the journal’s editorial process over the past 12 months. In 2018, a total of 186 papers were published in the journal, with a median time to first decision of 16 days and a median time to publication of 40 days. The editors would like to express their sincere gratitude to the following reviewers for their cooperation and dedication in 2018:
Abuzaid, AhmedBai, RenrenAdamus-Białek, WiolettaBajpai, RichaÅhman, IngerBajpai, VivekAkbar, MohammedBak, Kathrine HolmgaardAkimov, Sergey A.Baran, JaroslawAl Zoughbi, WaelBaran, AnnaAlhouayek, MireilleBarandiaran, IratiAlloza, IraideBarber, TeresaÁlvarez, María S.Basak, DebasishAndreev, KonstantinBasak, SujitAngeles-Boza, AlfredoBazire, AlexisAnifandis, GeorgeBell, Douglas A.Annunziata, OnofrioBellosta, PaolaAnsari, FarhanBellstedt, PeterAnsquer, Jean ClaudeBernardi, PaoloAntonov, IvanBesalú, EmiliAraniciu, CătălinBhattacharjee, SonaliAroor, AnnayyaBhattacharya, SupriyoAssaf-Balut, CarlaBian, LiAtobe, MasakazuBisson, JonathanAvnet, SofiaBiswas, SantanuAyciriex, SophieBlair, LauraBackert, SteffenBłaszczyk, JarosławBacolla, AlbinoBollati-Fogolín, MarielaBader, OliverBonnet, VeroniqueBadrising, UmeshBorchman, DouglasBorgia, AlessandroDachs, GabiBorota, AnaDaniellou, RichardBörries, MelanieDash, BirajaBose, DebojitDe Laureto, Patrizia PolverinoBossi, GianlucaDe Rosa, Maria CristinaBranza-Nichita, NoricaDechant, ReinhardBrasó-Maristany, FaraDel Giudice, RitaBreitenfeld Granadeiro, LuizaDellis, OlivierBreunig, HansDeLuca, JenniferBridel, ClaireDenkova-Kostova, RositsaBrumeanu, Teodor-DoruDepalma, Ralph G.Bruzzone, SantinaDeryusheva, SvetlanaBucki, RobertDeshpande, RahulBucova, MariaDeshpande, ShayuBurger, MichaelDi Donato, MarziaBurghardt, KyleDi Fazio, PietroButtstedt, AnjaDi Mauro, Maria DomenicaBuxton, DenisDianzani, IrmaCaballero, JulioDimova, ElitsaCairrão, ElisaDing, JianxunCalcutt, MichaelDistel, MartinCammas-Marion, SandrineDittmer, Neal T.Caprio, MassimilianoDoddapattar, PrakashCarabineiro, SoniaDomínguez Rebolledo, Álvaro EfrénCarta, GianfrancaDong, YiboCaruso, GianlucaDonno, DarioCava, ClaudiaDrutovic, DavidChai, Han-haDu, DeguoChalla, DilipDuan, WeiChatgilialoglu, ChryssostomosDubbeldam, JohanChavez-Santoscoy, Rocio AlejandraDuffy, TomasChen, Chun-JungDurrant, Jacob D.Chiaramello, AnneDusi, VeronicaChin, MichaelDuwaerts, CarolineChoi, Ucheor B.Dziekońska-Kubczak, UrszulaChua, WinnieEgel, RichardCojoc, DanEid, NabilColoff, JonathanEl Ghoch, MarwanCompton, AlisonEleftheriadis, TheodorosCoppotelli, GiuseppeElgamel, AliCosta, MarinaElliott, Hannah R.Creuzenet, CaroleEnderlein, JörgCronin, LeeEngelhart, AaronEnguita, FranciscoGroßhans, JörgEspen, LucaGruengreiff, KurtEwing, AilithGuedes, Rita C.Fach, L. MatthiasGuerra, CarmenFalahati Anbaran, MohsenGupta, MohanFalasconi, IreneGutierrez-Aguilar, ManuelFan, WeizhengHakenberg, JörgFeldman, Steven R.Halquist, MatthewFelgueiras, HelenaHamada, MichiakiFernandes, Maria J.Han, WeiweiFerreira, JoanaHanrieder, JörgFiorucci, StefanoHardwick, James P.Flavell, RobertHaro, IsabelFloresta, GiuseppeHashimoto, TakashiFrancis, HaneHassan, Sherif T. S.Frolov, AndrejHausch, FelixFukuchi, SatoshiHe, HailunFuller, AmeliaHe, HongliangFuxreiter, MonikaHenen, Morkos A.Gabrielska, JaninaHengesbach, MartinGagat, MaciejHibner, GrzegorzGaligniana, MarioHillard, Cecilia J.Gallo, MonicaHiroaki, HidekazuGambari, LauraHirota, KiichiGarcia-Sosa, Alfonso T.Hodakoski, CindyGase, KlausHoefer, ImoGavenonis, JasonHolley, CristopherGelinas, AmyHooda, JagmohanGerbino, AndreaHrabal, RichardGetahun, AndrewHsin, Kun-YiGeuns, Jan M.C.Huang, ChunfaGhislat, GhitaHuling, JenniferGillet, ValHumphrey, TimGood, Deborah J.Hwang, Dae YounGorman, AdrienneIadevaia, ValentinaGoroncy, AlexanderIbba, MichaelGowda, Siddabasave B.Ide, KazukiGraber, Zachary TIkawa, YoshiyaGrabinska, KarionaIm, Chang-NimGradinaru, Andrei CristianInglot, TadeuszGreen, BenInoue, KazushiGrisoni, FrancescaIohara, DaisukeGrkovic, TanjaIvanov, IvanIvanov, Alexander V.Kocsis, BelaJamshed, ShaziaKoirala, NiranjanJaradat, NidalKojima-Yuasa, AkikoJaworski, KrzysztofKõks, SulevJenssen, HåvardKothe, UteJi, ChengKotula-Balak, MalgorzataJiang, Qiu-XingKreider, RichardJiang, QingKristensen, MieJicsinszky, LászlóKrogh, NicolaiJiménez-Movilla, MariaKu, SeockmoJirkovsky, EduardKucharikova, SonaJochum, ChristophKuppusamy, ManiselvanJohnson, Jason L.Kurrikoff, KaidoJones, KarenKushner, Sidney R.Juretić, DavorKuwahara, MasayasuJust, SteffenLachowicz, Joanna IzabelaK A Foyez, MahmudLafontaine, DenisKale, AbhijitLagerstedt, Jens O.Kanazawa, MasatoLah, JurijKarakas, MahirLan, LanKasperkiewicz, PaulinaLavogina, DarjaKataoka, NaoyukiLayek, BuddhadevKathuria, HimanshuLe Hir, RozennKato, Takamitsu A.Lebar, MatthewKestler, HansLee, Chia-HwaKevadiya, BhaveahLee, Hee-JoKhadka, ManojLee, Joo HyoungKhadka, ManojLee, BethKhokhlatchev, Andrei V.Lei, WeiKhurshid, ZohaibLeverett, BetsyKim, Kyung BoLi, Wen-WuKing, Oliver D.Li, YanKirberger, MichaelLi, ZidaKirpich, Irina A.Li, GuohuiKiszonas, Alecia M.Libich, DavidKitajima, HidetoshiLilleker, JamesKlar, AgnesLin, QishanKlebe, GerhardLin, GangKlein, Gordon L.Lin, Ruo-KaiKnittelfelder, OskarLin, Long-LiuKnoell, RalphLindberg, GerrickKnoshaug, EricLinnstaedt, Sarah D.Koch, WojciechLippens, GuyLiu, WeiMerk, DanielLiu, CongMerkle, DanielLiu, YongqiangMichels, Alexander J.Liu, ZiqingMilano, FrancescoLizardi-Mendoza, JaimeMills, KerryLoewendorf, Andrea I.Mironescu, MonicaLoizides-Mangold, UrsulaMishra, Rupesh K.López-Andrés, NataliaMitchell, ToddLorberboum-Galski, HayaMohamedali, KhalidLorenz, KristinaMolina, Patricia E.Lu, JunMonsarrat, PaulLukyanov, PavelMooers, BlaineMa, ZhikunMoores, CarolynMacín, Stella MarisMor, AmramMaeder, Micha TobiasMorales-Tirado, VanessaMagan-Fernandez, AntonioMorikawa, ToshioMaher, PamelaMorita, YujiMaier, ClaudiaMorris, JamesMallipeddi, Prema LathaMozos, IoanaMandal, Mihir KumarMukaida, NaofumiManfrin, C.Mukherjee, SomnathManickam, NandiniMuresan, VladManjunath, ManuboluMurphy, ShonaManninger, MartinMurray Stewart, TracyMarinelli, LisaMuschiol, JanMariscal, VicenteNagy, LauraMartin, KeithNandi, SaikatMartínez-García, MarcosNapolitano, FrancescoMartinez-Mayorga, KarinaNarayan, PrakashMartinod, KimberlyNarayanaswami, VasanthyMartins, NatáliaNasir, OmaimaMasulli, MariaNawaz, MuhammadMatsumoto, YasuhikoNawaz, MuhammadMazzilli, FernandoNaydenov, StefanMcCarthy, PumtiwittNeto, João Ruggiero BrandaoMcClements, LanaNeumann, KonstantinMcMurry, JonathanNeves, VeraMcPhillie, MartinNiedziela, TomaszMedbury, Heather JeanNielsen, HenrikMehta, PoojaNimalaratne, ChamilaMeier, Kathryn E.Noda, TakeshiMenand, BenoîtNybo, EricMendoza-Mendoza, ArtemioObšil, TomášO’Gallagher, KevinPocheć, EwaOgunjirin, AdebowalePohl, PeterOhshiro, TakashiPoole, David C.Ojima, KoichiPoon, IvanOliva, RominaPopa, ValentinOri, OttorinoPope, ChadOrsolya, TőkePosselt, GernotOta, MotonoriPossik, EliteOtani, KeisukePoudel, BarunOthumpangat, SreekumarPowrózek, TomaszOvervad, Thure FilskovPrimus, Carolyn M.Pal, KasturiPulvirenti, AlfredoPalamà, IlariaPyzik, MichalPalermo, EdmundQuelle, DawnPan, YangangRafiei, HosseinPan, MingguiRagg, EnzioPanaccione, DanielRajpurohit, SurendraPandolfini, TizianaRamasamy, ElamparuthiPannerselvam, SureshRaspotnig, GüntherPaplińska-Goryca, MagdalenaRauch, BernhardPappa, AglaiaRegalado-González, CarlosParajuli, SarojReissmann, SiegmundParashar, DeepakRemesh, SoumyaParrettini, SaraRen, HangPatrice, ThierryRentero, CarlesPavon-Djavid, GracielaRequicha, JoãoPeeters, Frederique E. C. M.Rescifina, AntonioPentzold, StefanRhee, Hyun-WooPereira, FlorbelaRicci, VittorioPerkins-Veazie, PenelopeRiccò, RaffaelePermyakov, EugeneRich, JeremyPerni, MicheleRodionov, RomanPestov, DimitriRoelen, BernardPietrocola, GiampieroRogozhin, EugenePietrzak, Aldona T.Roh, ChanghyunPilgrim, David B.Roscini, LucaPilz, StefanRoy, DipankarPires, DouglasRozhdestvensky, TimofeyPires, Regina HelenaRubattu, SperanzaPlemper, RichardRuelland, EricPletzer, DanielRusso Krauss, IrenePoad, BerwyckRutkovskiy, ArkadyPoater, JordiRuzek, DanielRybakowska, IwonaSingh, RajbirRydz, JoannaSiniscalco, DarioSadovskaya, IrinaSipos, FerencSaito, RyokoSkatchkov, SergueiSaito, KosukeSkendi, AdrianaSalinas Rodríguez, BeatrizSliwinska, AgnieszkaSamsoondar, JoshuaSmani, YounesSamwel Nyandoro, StephenSmith, Tim A. D.Sanak, MarekSmolka, MarcusSanchez-Martinez, MelchorSobotta, ŁukaszSanchez-Mut, Jose V.Sodt, Alexander J.Sang, Bing OngSong, JiajiaSankaranarayanan, Nehru VijiSong, JaewhanSanmartí, RaimonSosic, IzidorSantulli, GaetanoSpeciale, AntonioSarathy, Vanessa V.Sreenath, BolisettySarkar, DhrubaSrivastava, AtulSarkies, PeterSrivastava, SaurabhSassi, YassineStadler, MarcSchaal, WesleyStathopulos, PeterSchettino, GiuseppeStout, RandySchmid, FriederikeStraus, Suzana K.Schneider, ClaudiaStuchlik, StanislavSchuster, Christopher F.Suddala, Krishna ChaitanyaScotti, MarcusSuh, Won HyukSelya, ArielleSun, JingjingSeo, Keun-SeokSun, HaoŠeva, JuricaSun, ChengjieSeverson, Aaron F.Sundar, ReshmaSgrignani, JacopoSyrén, Per-OlofShaw, SubrataTa, HangShen, YiTacconelli, StefaniaSheri, MadhuTachikawa, MasashiShilton, BrianTailhades, JulienShimizu, ToshiyukiTakahashi, KoretaroShiraishi, TakehikoTakahashi, HidehisaShukla, Sanjay K.Takashiba, ShogoSidhu, RohiniTakegahara, NorikoSil, PayelTalora, ClaudioSilva, Pedro JorgeTalukder, PoulamiSimoes, ZilaTanaka, NobuyukiSinger, Mitchell H.Teotia, PoojaSingh, AnilTerekhova, Irina V.Teschke, RolfWang, YongqiangThompson, Karl ManleyWarta, RolfThorner, JeremyWarwicker, JimThyparambil, AbyWebb, ChailleTiefenauer, LouisWebb, Samuel J.Tiwari, Rakesh K.Wei, ChangyongTofani, DanielaWeigand, Julia E.Tomanek, BoguslawWeikl, ThomasTomizaki, Kin-yaWeisman, RonitTribulova, NarcisaWen, JiaTriebl, AlexanderWen, Jia DiTrivedi, DarshanWestbrook, JohnTroppmair, JakobWhitfield, PhilTsentalovich, YuriWigneshweraraj, SivarameshTyteca, DonatienneWillerth, Stephanie M.Upadhyay, Rakesh K.Winkler, AndreasUrbanelli, LorenaWinterhalter, MathiasUsui, KenjiWinters, JeffreyVan Der Vorst, EmielWong, Sek-ManVan Laar, TriciaWong, Boon SengVarnek, AlexandreXiang, DongxiVasauskas, Audrey A.Xu, ZhijieVassalle, CristinaYadav, MarshleenVelkov, TonyYamashita, AkiraVenturini, CristinaYang, XiaochunVerardi, AlessandraYang, Deng-HoVergés, MarcelYang, Jae-WonVerma, RajniYeger, HermanVezzu, DileepYin, JianVicente Manzanares, MiguelYu, GangVillar-Piqué, AnnaYunusa, IsmaeelVlachou, MarilenaZakrocka, IzabelaVora, MehulZhang, YongliVosegaard, ThomasZhang, CaiguoWakabayashi, SadaoZhao, ZongminWallace, DanielZhao, XuebingWall-Medrano, AbrahamZhao, BaoyinWan, ShibiaoZhen, XuechuWang, QiuanZhou, ZhanxiangWang, PengchengZloh, MireWang, BaominZorbas, ChristianeWang, XiaoboZou, WeiWang, WenjingZüllig, RichardZüllig, ThomasZuvela, PetarZuluaga, Paola
Zur, Krzysztof Kamil


